# Investigating the effects of comprehensive smoke-free legislation on neonatal and infant mortality in Thailand using the synthetic control method

**DOI:** 10.1016/j.eclinm.2020.100560

**Published:** 2020-10-02

**Authors:** Márta K. Radó, Frank J. van Lenthe, Aziz Sheikh, Jasper V. Been

**Affiliations:** a Division of Neonatology, Department of Paediatrics, Erasmus MC – Sophia Children's Hospital, University Medical Centre Rotterdam, PO Box 2060, 3000 CB Rotterdam, the Netherlands; b Department of Public Health, Erasmus MC, University Medical Centre Rotterdam, Rotterdam, the Netherlands; c Asthma UK Centre for Applied Research, Usher Institute, The University of Edinburgh, Edinburgh, UK; d Centre of Medical Informatics, Usher Institute, The University of Edinburgh, Edinburgh, UK; e Division of General Internal Medicine and Primary Care, Brigham and Women's Hospital/Harvard Medical School, Boston, MA, USA; f Department of Medicine, Harvard Medical School, Boston, MA, USA; g Department of Obstetrics and Gynaecology, Erasmus MC, University Medical Centre Rotterdam, Rotterdam, the Netherlands

**Keywords:** Smoke-free legislation, Infant mortality, Child health, Thailand, Synthetic control method

## Abstract

**Background:**

Almost all of the evidence on the benefits of smoke-free legislation on child health comes from evaluations in high-income countries. We investigated the effects of Thailand's 2010 comprehensive smoke-free legislation on neonatal and infant mortality.

**Methods:**

To overcome some of the methodological issues inherent to traditional quasi-experimental methods, we applied the novel synthetic control approach. Using 2001–2017 country-level panel data from the World Bank and Penn World datasets, we estimated the effects of smoke-free legislation as the difference between the outcome trends in Thailand versus those in a synthetic control country. The synthetic control country was composed of ‘control’ middle-income countries without comprehensive smoke-free legislation to recreate trends in Thailand in the 2001–2009 pre-legislation outcomes and covariates. We compared the legislation effects to ‘placebo effects’ obtained for each control country by fictitiously assuming that comprehensive smoke-free legislation was introduced there in 2010, similar to Thailand.

**Findings:**

Neonatal and infant mortality decreased by 2.9% and 2.8%/year respectively following smoke-free legislation, with an estimated 7463 infant deaths (including 4623 neonatal deaths) having been averted over eight years. The results were robust to different specifications of the control countries. Comparison with placebo effects indicated that the findings were unlikely to be attributable to factors other than the smoke-free legislation.

**Interpretation:**

Expanding comprehensive smoke-free policies to middle-income countries can support national efforts to achieve Sustainable Development Goal 3.2 for reducing preventable early-life deaths.

**Funding:**

Netherlands Lung Foundation, HDRUK, Asthma UK center for Applied Research and NIHR Global Respiratory Health Unit (RESPIRE).

**Research in context**

**Evidence before this study**

Tobacco use is the leading preventable cause of mortality and morbidity globally, and children are especially vulnerable. There is a growing body of literature supporting positive effects of smoke-free legislation on child health; according to our 2017 systematic review smoke-free legislation was associated with substantial reductions in preterm birth (by 3.8% [95% CI 1.2 to 6.·4]), hospital attendance for asthma (by 9·.8% [95% CI 3.0 to 16.·6]), and hospital attendance for lower respiratory tract infections (by 18·.5% [95% CI 4.2 to 32.·8]). In England smoke-free legislation was furthermore associated with a significant reductions in neonatal mortality by 7·6% (95% CI 3·4 to 11·7) and infant mortality by 6·3% (95% CI 2·9 to 9·6). However, the generalizability of these findings to low- and middle-income countries is unclear since in such countries, high background outdoor and indoor air pollution, low awareness of tobacco-related harm, and poor economic conditions might suppress the positive effects of smoke-free legislation. In a recent study, implementation of comprehensive smoke-free laws in Brazil was associated with an immediate 5·2% (95% CI 2·1 to 8·3) and an additional annual 0·4% (95% CI 0·1 to 0·7) decrease in infant mortality and an immediate 3·4% (95% CI 0·1 to 6·7) decrease in neonatal mortality.

**Added value of this study**

We found that the child health benefits of smoke-free legislation in Thailand were comparable to those found in high-income countries. Our study confirms the effectiveness of smoke-free legislation to promote neonatal and infant survival in a middle-income setting where previously only very limited research was conducted. To our knowledge, this is the first assessment of the impact of tobacco control legislation on child health using the robust synthetic control method which provided further robust evidence that the confirmed link between smoke-free legislation and child health is indeed causal.

**Implications of all the available evidence**

Taken together, the available evidence indicates that smoke-free legislation can indeed also bring about substantial child health benefits in middle-income countries. Tobacco control measures are particularly important in less developed regions where the tobacco industry retains a very influential role. The findings from this study support the need for further acceleration of the implementation of smoke-free policies across the globe.

Alt-text: Unlabelled box

## 1 Introduction

Tobacco smoke exposure during perinatal and early life increases the risk of stillbirth, preterm birth, low weight birth, and neonatal and infant death [1], [2], [3]. Annually, approximately 166,000 avoidable deaths and 6·6 million disability-adjusted life years (DALYs) among children can be attributed to second-hand smoke exposure worldwide [4,5]. As a consequence, the protection of children from the harm of tobacco smoke exposure has been identified as a vital tool to achieve the United Nations’ Sustainable Development Goals 3.2.1 and 3.2.2, aimed at improving under-five and neonatal mortality rates [6].

Smoke-free policies have the potential to reduce tobacco smoke exposure during pregnancy and infancy, and through so doing reduce the risk of a number of serious adverse health outcomes [7]. Based on a recent systematic review and meta-analysis, implementation of smoke-free legislation covering enclosed public places was associated with a 3·8% (95% confidence interval [CI] 1·2 to 6·4) decrease in preterm birth rates, a 9·8% (95% CI 3·0 to 16·6) decrease in rates of hospital attendance for asthma exacerbations, and an 18·5% (95% CI 4·2 to 32·8) decrease in rates of hospital attendance for lower respiratory tract infections [7]. Furthermore, evidence from England indicated that smoke-free legislation reduced neonatal mortality by 7·6% (95% CI 3·4 to 11·7) and infant mortality by 6·3% (95% CI 2·9 to 9·6) [8].

At present, the evidence on the early-life health impact of smoke-free legislation is almost entirely derived from evaluations in high-income countries (HICs) [7,9]. These findings may not easily be generalized to low- and middle-income countries (LMICs) for a number of reasons – for example, outdoor air pollution, indoor air pollution due to biomass used for cooking and heating, household tobacco smoke exposure, and tobacco smoke exposure among pregnant women are significantly higher, whereas awareness of tobacco-related harms is significantly lower in LMICs than in HICs [10], [11], [12] Moreover, pregnancy outcomes are generally poorer in LMICs than in HICs due to reduced antenatal care capacities [13]. We are aware of only one study that investigated the effectiveness of smoke-free legislation on infant health from a middle-income country (MIC), namely Brazil [9]. Based on this study, the implementation of smoke-free laws across Brazil was estimated to have prevented 15,068 infant deaths over 12 years [9].

Our study extends the scope of research to Thailand, a MIC from the East Asian and Pacific region. Thailand has been a tobacco control leader in the region for a long time [14,15]. Partial smoke-free legislation came into practice in Thailand in 1992, prohibiting smoking in public places where such legislation can easily be implemented such as hospitals, schools, and air-conditioned workplaces (Fig. S1) [16,17]. However, it still allowed smoking in non-air-conditioned public areas and workplaces. In 2010, the legislation was extended to become comprehensive as advocated by the World Health Organization (WHO), prohibiting smoking in all indoor public places, indoor workplaces, public transport (except international airports where designated smoking areas are available), and some outdoor areas [5,18,19]. Previous studies found that the comprehensive smoke-free legislation was enforced well (especially in case of indoor workplaces, indoor public places, and restaurants) [20,21]. Compliance was rated in 2013 to be 7/10 based on a WHO evaluation [22]. Further, the implementation of smoke-free legislation was followed by a gradual decrease in smoking prevalence (i.e. from 21·7% in 2010 to 19·9% in 2016) [23] and in second-hand smoke exposure at home or enclosed public places among youth (i.e. from 68% in 2009 to 39% in 2015) [24].

We applied a novel synthetic control method to estimate the effects of Thailand's introduction of comprehensive smoke-free legislation on neonatal and infant mortality. This method recreates the counterfactual scenario (i.e. what would have happened if smoke-free legislation had not been implemented) as a weighted average of multiple control countries that did not introduce comprehensive smoke-free legislation during the study period [25,26]. The advantage of this method, when compared to other quasi-experimental methods, is that it provides a better counterfactual scenario (i.e. it recreates better the pre-legislation trends in Thailand), allowing less biased estimation [25], [26], [27].

## 2 Methods

### 2.1 Study design and setting

Our study covered the pre-legislation period between 2001 and 2009 and the post-legislation period 2010 to 2017. Data from before 2001 were not included to ensure that any impact of the 2001 "30 Baht" Thai healthcare reform on underlying trends in neonatal or infant mortality would not distort our estimation [28]. First, we estimated how neonatal and infant mortality statistics would have progressed in Thailand if the smoke-free legislation had not been implemented. The synthetic control method estimates this counterfactual scenario by constructing a synthetic control country as a combination of available control countries. In our case, the synthetic control country was the weighted average of other MICs with available data that did not implement comprehensive smoke-free legislation until the end of the observation period (2017), but had been otherwise similar to Thailand pre-legislation (Table S1). Thereafter, we estimated the impact of the smoke-free legislation as the difference between temporal patterns in the outcomes between Thailand and its synthetic control country post-legislation.

### 2.2 Variables and data sources

The set of available control countries used for constructing the synthetic control country is referred to as the donor pool. We selected countries for the donor pool that did not implement comprehensive smoke-free legislation until the end of the observation period (2017) according to the WHO Report on the Global Tobacco Epidemic 2019 [5]. WHO defines national smoke-free legislation as comprehensive if it prohibits smoking in indoor workplaces and indoor public places, including health-care facilities, educational facilities, government facilities, public transports, bars, and restaurants, without allowing designated smoking rooms [29]. The donor pool should resemble the main characteristics of Thailand in order to avoid interpolation and extrapolation bias (i.e. bias arising when there is not sufficient overlap between the control and intervention groups in their outcomes) [30,31]. Thus, we restricted the donor pool to MICs according to the World Bank's Country Classification System [32]. After omission of 12 countries with missing values in the predictor or outcome variables (Table S2), 49 countries constituted the final donor pool.

Table 1 provides an overview of the outcome and predictor variables, all of which were obtained annually. Data on the outcome variables – national-level neonatal mortality and infant mortality – were extracted from the World Bank Database for the 2001–2017 observational period [33]. We selected predictor variables based on their impact on the outcomes, as shown in previous work [30,34]. Consequently, we controlled for variables that capture the social and economic status of the countries, quality and access to healthcare facilities, and air pollution. Additionally, we also used as predictors the pre-legislation values of the outcome variables. Predictors were collected from the World Bank Database, except for ‘Openness to trade’ which was obtained from the Penn World Dataset [35].

**Table 1:** Description of the outcome and predictor variables.

Variables		Database	Definition
Outcome variable	Neonatal mortality	World Bank Database (2001–2017 period)	The number of neonates who die within 28 days after birth per 1000 livebirths
	Infant mortality	World Bank Database (2001–2017 period)	The number of deaths among infants younger than one year of age per 1000 livebirths
Social and economic variables	GDP	World Bank Database (2001–2009 period)	Gross domestic product per capita (PPP)
	Rural population	World Bank Database (2001–2009 period)	The proportion of the population living in rural areas as defined by national statistical offices
	Female primary education completion rate	World Bank Database (2001–2009 period)	The ratio of the number of new female entrants in the last grade of primary education (regardless of age) and the number of females at the entrance age for the last grade of primary education
	Fertility rate	World Bank Database (2001–2009 period)	The average number of children born to a woman (given women survive the childbearing age and fertility is in line with age-specific fertility rates of the specified year)
	Openness to trade	Penn World Dataset (2001–2009 period)	The share of exports plus imports compared to nominal GDP
Health-care indicators	Health expenditure	World Bank Database (2001–2009 period)	Current health expenditure per capita expressed in international dollars (PPP)
	Hospital beds	World Bank Database (2001–2009 period)	The number of hospital beds available per 1000 people
	Drinking water	World Bank Database (2001–2009 period)	The proportion of the population with access to basic drinking water (i.e. collection time < 30 min)
Air pollution	Clean cooking	World Bank Database (2001–2009 period)	The proportion of the population with access to clean fuels and technologies for cooking
	CO_2_	World Bank Database (2001–2009 period)	Carbon dioxide emissions in kiloton

Abbreviations: GDP= Gross domestic product; PPP= Purchasing power parity; CO_2_= Carbon dioxide.

### 2.3 Constructing the synthetic control countries

We constructed two synthetic control countries: one reproducing the pre-legislation trend in neonatal mortality, the other mirroring the trend in infant mortality. Each synthetic control country was constructed as a weighted average of countries from the donor pool, as follows.

Let *N* be the number of countries, n=1is Thailand and *n* ≥ 2 are the countries in the donor pool, $Y_{n}^{t}$ denotes the outcome properties (i.e. neonatal or infant mortality) of the *n* country in *t* year, and $W_{n}^{Y}$refers to the weight of *n* country in constructing the synthetic control country [25,26,30]. The $Y_{synth}^{t}$ outcome variables at *t* year in a synthetic control country can be calculated as the weighted average of the countries in the donor pool (Eq. (1)).

(1) $$Y_{synth}^{t}=\sum_{n=2}^{N}W_{n}^{Y}*Y_{n}^{t}$$

The $W_{n}^{Y}$ country weights were the result of the optimization that ensures that Thailand and the synthetic control country were as similar as possible in a set of $X_{n}^{t}$ pre-legislation predictors [25,26,30]. This algorithm took into account that not all predictors contributed equally to a given outcome variable (e.g. the lagged value of infant mortality better predicted current infant mortality than the proportion of the population living in a rural area did, Table S3). We applied the default optimization method that minimalized the root mean squared prediction error (RMPSE) for the pre-legislation period (Eq. (S1)) using Broyden, Fletcher, Goldfarb, and Shanno's (BFGS) and quasi-Newton algorithms [36]. We built the synthetic control countries using the Synth package, which runs in the R environment [36].

### 2.4 Estimating the legislation effect

After constructing the synthetic control countries based on the pre-intervention trends, we assessed the $\alpha_{t}^{Y}$ dynamic legislation effect in *t* year quantified as the post-legislation difference in *Y* outcome variable of Thailand and that of the synthetic control country:

(3) $$\alpha_{t}^{Y}=Y_{1}^{t}-Y_{synth}^{t},$$

where $Y_{synth}^{t}$ is defined in Eq. (1).

To obtain the percentage change in the outcome associated with the legislation for each post-legislation year, we divided the $\alpha_{t}^{Y}$ legislation effect by the outcome of the synthetic control country in *t* year. Further, we calculated the year-specific numbers of avoided deaths as the product of $\alpha_{t}^{Y}$ legislation effect in *t* year and the year-specific number of live births (i.e. crude birth rate per 1000 multiplied by the total number of population as derived from the Word Bank Database). The sum of the year-specific avoided deaths quantifies the total legislation effect.

### 2.5 Placebo test

We conducted ‘placebo tests’ that compared the legislation effect to the ‘placebo effects’ that were obtained for each control country by fictitiously assuming that comprehensive smoke-free legislation was introduced there at the same time as in Thailand (i.e. 2010). This test allowed us to assess whether the estimated $\alpha_{t}^{Y}$legislation effect was indeed most likely a response to the legislation or whether it could be merely the result of other (i.e. confounding or co-intervention) processes.

The placebo effects were estimated in three steps [26]. First, we constructed a synthetic ‘control country’ for every control country from the donor pool using the same predictors and pre-legislation period as for constructing a synthetic control country for Thailand. Second, we excluded from further consideration those countries for which we could not find a synthetic control country that reproduced well the outcomes in the pre-legislation period (2001–2009). Two exclusion criteria were used in separate analyses: we discarded countries with pre-legislation RMPSE higher than 0·03 or higher than in Thailand (i.e. 0·14 for neonatal mortality and 0·19 for infant mortality). Third, for each remaining *k* country, we defined the $\omega_{tk}^{Y}$ placebo effect as the difference between the *Y_k_* outcome of the *k* control country and $Y_{synth_{k}}^{t}$ of its synthetic ‘control country’.

(4) $$\omega_{tk}^{Y}=Y_{k}^{t}-Y_{synth_{k}}^{t}=Y_{k}^{t}-\sum_{n=1,n\neq k}^{N}W_{nk}^{Y}*Y_{n}^{t},\;\;\text{where}\;\;k\neq 1$$

### 2.6 Sensitivity analyses

We explored the robustness of our analyses by undertaking four sets of sensitivity analyses using different donor pools. First, we tested the effect of being more restrictive with economic proximity and narrowed down the donor pool to only upper-MICs (according to the World Bank's Country Classification System) [32]. In this case, we restricted the analysis to 28 eligible control countries (five countries were not included due to missing data, Table S2). Second, we defined the donor pool based on geographical proximity to Thailand instead of using economic proximity. More specifically, we selected 12 countries from the East Asian and Pacific region (12 countries from this region were not included due to missing data, Table S2). Third, we removed from the donor pool the control country that received the highest weight in creating the synthetic control country in the main analysis to test whether the results of the main analysis could be merely attributed to processes (e.g. health care reforms, the introduction of new vaccines, epidemics) in this important control unit [37]. Fourth, we reduced the donor pool to countries that did not have any smoke-free legislation (not even partial) during the study period.

Further, we assessed sensitivity of our results to excluding donor countries base on missing data. We used the imputeTS package (using interpolation function) to impute missing values [38], retaining 53 countries and omitting 9 countries where interpolation was not possible due to less than three observations in predictors or due to missing values in the outcome variable. Finally, we conducted a sensitivity analysis controlling for annual cigarette consumption at the country-level. As comparative and historical smoking prevalence data were not available, we used estimated cigarette consumption data from tobacco sales data (36 countries were omitted due to missing data) [39].

### 2.7 Ethical considerations

This study did not require ethical approval since only aggregated and publicly available data were used.

### 2.8 Role of the funding source

The funders of the study had no role in study design, data collection, data analysis, data interpretation, or writing of the report. The corresponding author had full access to all the data in the study and had final responsibility for the decision to submit for publication.

## 3 Results

**Fig. 1:**
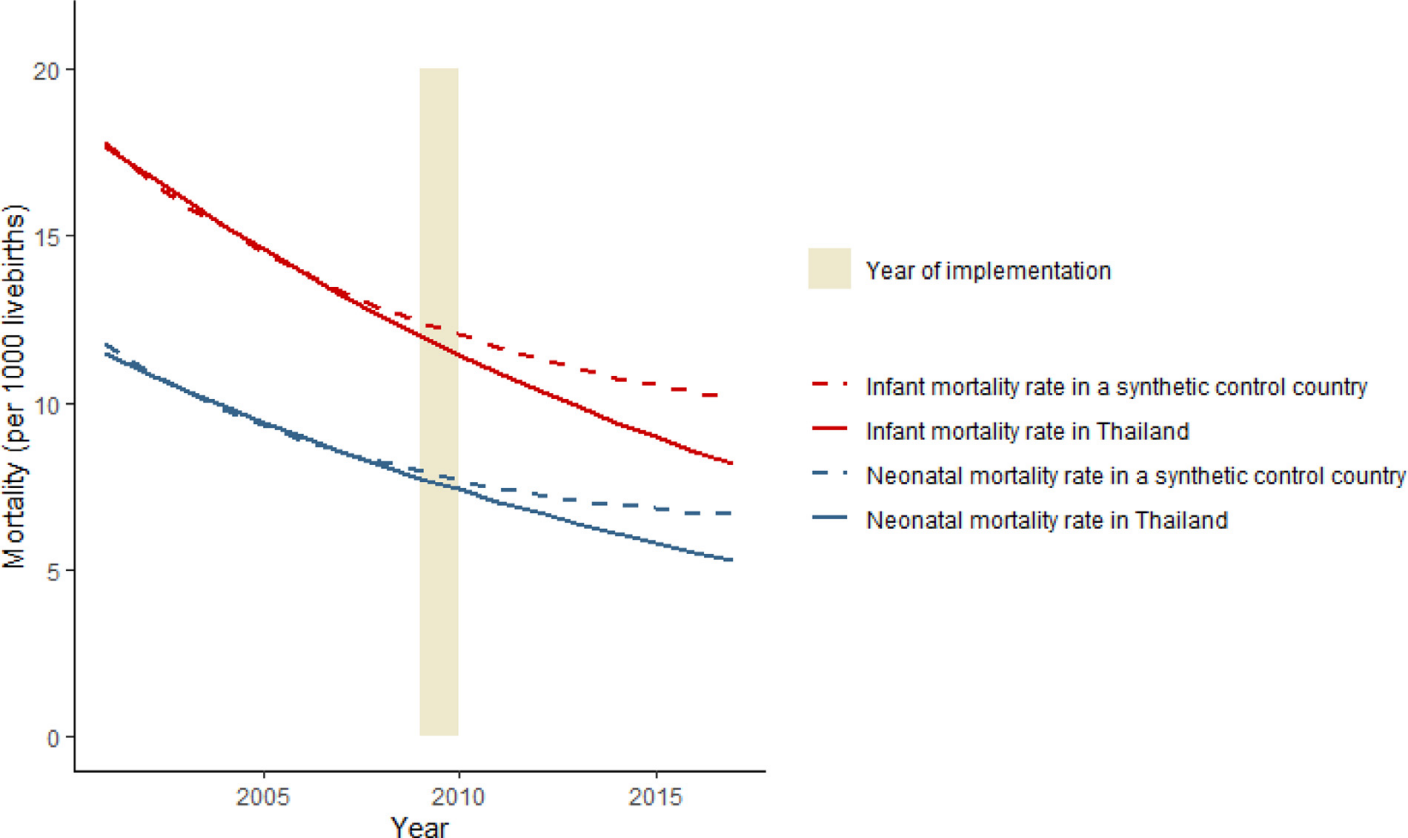
Trends in neonatal and infant mortality rate: Thailand versus the synthetic control country (2001–2017).

Table 2 displays the selected control countries and their respective weights contributing to the synthetic control countries. For reproducing the pre-legislation neonatal mortality trend in Thailand, the synthetic control country was the weighted average of Malaysia, Nicaragua, Moldova, China, and Bhutan (listed according to their weights). The infant mortality trend in Thailand was replicated as a weighted combination of Malaysia, Nicaragua, Moldova, Sri Lanka, China, Bangladesh, and Morocco. The weighted average of the control countries adequately reproduced the pre-legislation average values of the predictor variables (Table 3) and the temporal trends in the outcomes of Thailand (Fig. 1).

**Table 2:** Country weights in the synthetic control countries for reproducing infant mortality and neonatal mortality trends in Thailand, 2001–2009.

Country^a^	Country weights	
	Neonatal mortality	Infant mortality
Malaysia	0·445	0·502
Nicaragua	0·364	0·264
Moldova	0·146	0·112
China	0·024	0·026
Bhutan	0·022	–
Sri Lanka	–	0·071
Bangladesh	–	0·024
Morocco	–	0·001

a This table contains only those control countries that contributed to the constitution of the synthetic control country with a larger than 0 weight.

**Table 3:** The mean values of the predictors across the pre-legislation period (2001–2009).

Variables	Thailand	Donor pool	Synthetic control country 1 (for neonatal mortality)	Synthetic control country 2 (for infant mortality)
*N* (number of countries)	1	49	5	7
Neonatal mortality (birth per 1000 livebirths)	9·49	20·02	9·53	8·84
Infant mortality (birth per 1000 livebirths)	14·70	34·90	16·24	14·75
GDP (PPP)	11392·68	7713·82	10303·71	11314·74
Health expenditure (PPP)	333·68	323·40	360·87	376·75
Clean cooking (proportion of the population)	70·19	55·22	70·72	70·50
Hospital beds (per 1000 people)	2·15	2·59	2·15	2·15
Drinking water (proportion of the population)	95·75	82·75	89·21	90·32
CO_2_ (kiloton)	238077·12	194627·53	213658·61	237615·41
Rural population (proportion of the population)	62·53	52·28	42·06	43·87
Female primary education completion rate (proportion of the population)	86·25	88·25	91·34	93·03
Fertility rate (average number of children)	1·58	3·13	2·31	2·30
Openness to trade (exports plus imports/nominal GDP)	143·53	90·85	140·15	143·40

Abbreviations: GDP= Gross domestic product; PPP= Purchasing power parity; CO_2_= Carbon dioxide.

Fig. 1 shows that there was a gradual divergence of the neonatal/infant mortality trends over time between Thailand and the control country in the 2010–2017 post-legislation period. Over this period, the relative reduction in neonatal mortality added up to 20·4% (on average 2·9% per annum) and that in infant mortality to 19·3% (on average 2·8% per annum). In absolute terms, this translates into an estimated aversion of 7463 infant deaths, including 4623 neonatal deaths, over eight years.

**Fig. 2:**
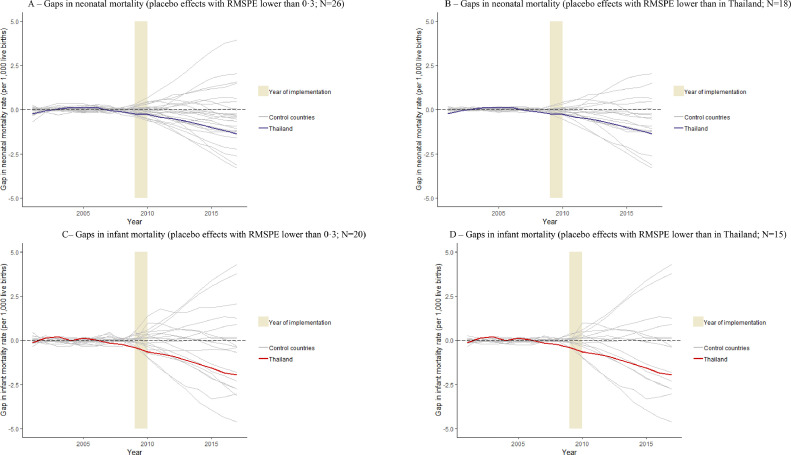
The estimated legislation effects in Thailand and placebo effects in middle-income countries that did not introduce comprehensive smoke-free legislation.

To assess whether our findings could have been influenced by other factors than the comprehensive smoke-free legislation, we estimated a placebo effect for each control country (Fig. 2A–D, Tables S4 and S5). The vast majority of control countries had a placebo effect that was smaller than the estimated legislation effect for Thailand. When we restricted the donor pool to those countries for which a very well-fitting synthetic control could be constructed (i.e. RMPSE was lower than the level that Thailand had; Fig. 2B and D), only three out of 18 countries had a larger placebo effect than the estimated legislation effect for Thailand in case of neonatal mortality and five out of 15 in case of infant mortality. To explore the potential underlying reasons for why some of the placebo effects were larger than the estimated treatment effect in Thailand, we performed a number of pre-specified sensitivity analyses.

**Fig. 3:**
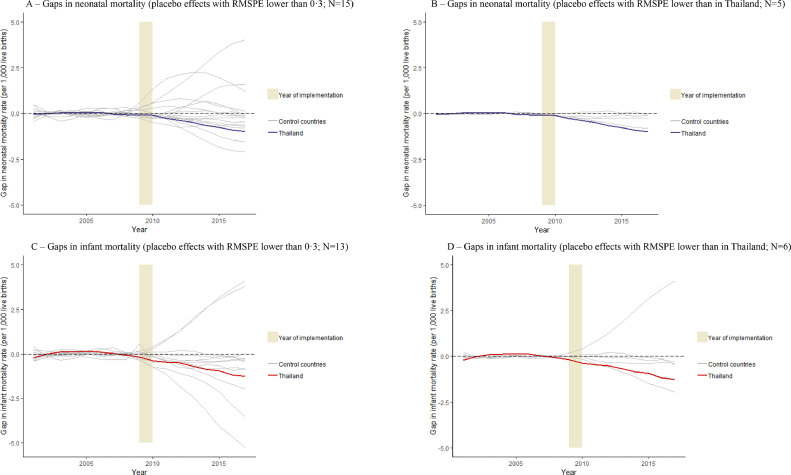
The estimated legislation effects in Thailand and placebo effects in upper-middle-income countries that did not introduce comprehensive smoke-free legislation.

**Fig. 4:**
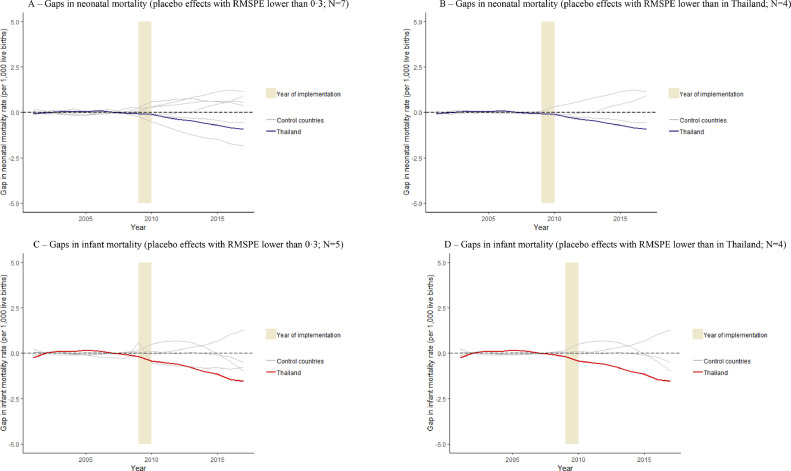
The estimated legislation effects in Thailand and placebo effects in countries from the East Asian and Pacific region countries that did not introduce comprehensive smoke-free legislation.

We restricted the donor pool to (1) upper-MICs (Fig. 3A–D and Table S6), and (2) countries from the East Asian and Pacific region (Fig. 4A–D and Table S7), (3) the donor pool without the country with the highest weight in the main analysis, Malaysia (Table S8 and Fig. S2), and (4) countries without partial smoke-free legislation (Table S9 and Fig. S3). All of these scenarios generated substantive legislation effects in Thailand, similar to the main analysis. Furthermore, when the donor pool was restricted to countries economically or geographically more similar to Thailand than in the main analysis, the legislation effects in Thailand were almost invariably larger than the placebo effects obtained from control countries (see Figs. 3 and 4). When we consider only those control countries for which a very well-fitting (i.e. RMPSE lower than the level that Thailand had) synthetic control was available (Fig. 3B, D and Fig. 4B, D), only Belize had a placebo effect that was slightly larger than the estimated treatment effect in Thailand, and only for infant mortality. Further, results were robust in sensitivity analyses in which we imputed missing values (Table S10 and Fig. S4) and accounted for cigarette consumption (Table S11 and Fig. S5).

## 4 Discussion

Using the novel synthetic control method for the first time in relation to the impact of smoke-free legislation on child health, we found that the 2010 national implementation of comprehensive smoke-free legislation in Thailand was associated with substantive decreases in neonatal and infant mortality (on average –2·9% and –2·8% per annum, respectively), translating to 7463 averted infant deaths (including 4623 averted neonatal deaths) over eight years. Placebo tests among countries similar to Thailand indicate that these changes were unlikely to be due to other factors. This indicates that smoke-free legislation is also likely to bring about positive changes in infant health in MICs, where there is currently a limited body of evidence to guide decision making [7,9].

The main strength of this study was the use of a synthetic control method that produces less biased estimations than other commonly used quasi-experimental methods [25,26,40,41]. The major challenge of estimating an intervention effect on observational data is the selection of appropriate control units. Most of the previously employed methods failed to adequately address this issue since they did not use any actual control units (e.g. used single-arm interrupted time series analysis) or used control units that deviated from the treatment unit prior to the introduction of the new legislation. Some previous studies attempted to adjust for pre-legislation differences between the control and intervention units using traditional regression methods (e.g. fixed-effect model), however, these methods are often subject to extrapolation or interpolation bias [25,26,42]. The synthetic control method provides a data-driven, transparent solution to the selection of appropriate control units. Further, the synthetic control method relaxes the linearity assumption of traditional longitudinal methods by allowing the difference in outcome between the treated and control countries to dynamically vary over time [25,26].

The synthetic control method offers the opportunity to assess whether the estimated effects may be attributed to processes other than the smoke-free legislation. We compared the effect of legislation in Thailand to the placebo effects that were obtained for each country of the donor pool by fictitiously assuming that a smoke-free law had been introduced in those countries. In the main analyses, a few placebo effects were stronger than the estimated effect in Thailand (in case of neonatal mortality: the Dominican Republic, Ghana, and Morocco; in case of infant mortality: Armenia, Bangladesh, Belize, Cameroon, and Ghana). These countries might display a larger placebo decrease than the actual decrease in Thailand due to various reasons. Armenia, for example, almost tripled its health expenditure in the post-legislation period [33]. The other exceptional cases typically occurred in lower-MICs, as opposed to upper middle-income Thailand, which might explain the more rapid improvements in infant mortality measures. After reducing the donor pool to countries that were economically or geographically closer to Thailand, we found that the legislation effect in Thailand always exceeded the placebo effects in the respective donor pools, except in case of infant mortality in Belize. However, Belize implemented (at the same time as the Thai smoke-free legislation, 2010) a successful hospital reform aiming to improve maternal and neonatal care which could explain their exceptional developments [43,44]. These results indicate that the observed improvements in neonatal and infant mortality in Thailand after 2010 as compared to the synthetic control country are unlikely to be related to factors other than the national introduction of comprehensive smoke-free legislation.

Our estimations were robust across different specifications of the donor pool. All sensitivity analyses showed a substantive reduction of neonatal and infant mortality in Thailand following comprehensive smoke-free legislation. Although these sensitivity analyses were useful in testing the sensitivity of our results to different model specifications, they permitted a less robust estimation than the main analysis due to a lower number of control countries. The donor pool was particularly reduced after the inclusion of cigarette consumption as a predictor. These data were unavailable for several important control countries, thus less appropriate control countries constituted the synthetic control country in this case. For example, Armenia was now included which likely explains the lower effect estimate as this country had tripled its health expenditure in the post-legislation period. Furthermore, the added value of this sensitivity analysis was low, as the cigarette consumption variable received a very low weight in creating the synthetic control country (<0·01%). Finally, a disadvantage of these data is that they did not incorporate illicit trade in tobacco sales and had low to moderate reliability for some LMICs [39].

Although we applied a novel and robust method, our study still had to rely on a quasi-experimental design since national policy interventions are typically not amendable to being evaluated using randomized control trials [27,45]. This approach, in general, limits researchers’ ability to rule out all residual confounding and potential influence of co-interventions. We are unaware of relevant national co-interventions in Thailand in the time period of that may have influenced our findings. Similar to other quasi-experimental studies, our long-term estimation might have been influenced by subsequent laws (e.g. a law requiring that health warnings cover at least 85% of the surface of cigarette packages introduced in 2014) [46].

In the light of the quasi-experimental nature of our study, it is important to consider circumstantial evidence supporting likely causality of our findings. First, the fact that smoke-free legislation was enforced well in Thailand increases the likeliness of it having an impact on smoking behavior and health outcomes [20,21]. Based on the WHO Tobacco Control Reports, initial compliance with the comprehensive smoke-free law was good (i.e. rated as 7/10 based on the 2013 WHO evaluation) [22]. Although compliance somewhat decreased in subsequent years (i.e. rated as 5/10 based on 2019 WHO report) [5], this was primarily due to reduced compliance with the legislation in bars, a public place rarely frequented by pregnant women and infants. As such, this decrease in the rating is unlikely to affect our estimation of the law's impact on early-life survival. Second, based on World Bank data, smoking prevalence steadily decreased from 21·7% in 2010 to 19·9% in 2016 [23], with Global Adult Tobacco Survey (GATS) data indicating that the decrease in the first year of the comprehensive smoke-free legislation occurred mainly among women [47]. Third, the Global Youth Tobacco Survey (GYTS) showed that between 2009 and 2015 second-hand smoke exposure at home or enclosed public places dropped dramatically among youth (i.e. from 68% to 39%) [24]. Decreased exposure to tobacco smoke exposure in the home via norm spreading is likely to be an important contributor to reducing overall second-hand smoke exposure among infants and pregnant women, hence benefiting neonatal and infant survival [48,49].

Despite achieving an overall good pre-legislation fit between Thailand and its synthetic countries, some deviation between them can be traced on the year before the intervention; a similar pattern was however also observed in a number of placebo tests. For Thailand, this initial divergence could occur due to an anticipation effect that has been described previously in other jurisdictions in relation to smoke-free legislation [50]. Further, partial smoke-free legislation was already in place even before the smoke-free law became comprehensive in 2010. However, this initial partial law was not well enforced based on a 2008 WHO evaluation, likely limiting its effectiveness [51]. Nevertheless, in the main analysis, we may have underestimated the true impact of a comprehensive law introduced against no background of any smoke-free policy, as Thailand already had limited smoke-free legislation in place. The sensitivity analysis comparing Thailand to countries without any smoke-free legislation (as opposed to also including countries with partial legislation) showed a larger impact on neonatal mortality than in the main analysis. This is in agreement with findings from Brazil, where the neonatal survival benefits were larger for comprehensive versus partial smoke-free laws [9]. However, these later results need to be cautiously interpreted due to a significantly reduced size of the donor pool. Finally, our study design was more limited compared to other studies that could select municipality-level units as compared to our country-level units or measure the outcomes on a monthly basis instead of our annual measurements [9].

Our results from Thailand concur with previous studies which were almost invariably conducted in HICs [7], although Thailand is embedded in a less favorable social, economic, and environmental setting. Although such aspects may have been anticipated to reduce the effectiveness of smoke-free legislation for example due to higher background levels of air pollution, other aspects such as compliance with and enforcement of the law, or cultural beliefs around smoking are also important and can be variable both across and within HIC and LMIC settings. The previous evidence indicates that smoke-free legislation decreases perinatal and infant tobacco smoke exposure, and in turn, improves infant health and averts a substantive number of infant deaths [7,52]. Our estimated legislation effects in Thailand are of similar magnitude in comparison to these earlier studies, as well as to a recent study conducted in Brazil [9]. By applying a novel and robust method, our findings strengthen the argument that the link between smoke-free policies and improvements in infant health is indeed causal and not merely due to inappropriate methodological approaches or contextual factors.

Future research should focus on capturing the effects of smoke-free legislation across multiple countries where such assessments are currently lacking. Recent developments in the synthetic control method may provide a robust vehicle to undertake this as this permits the simultaneous observation of multiple ‘treated’ countries [53], [54], [55] Finally, future studies should also extend the scope of research to assess effectiveness of extending smoke-free policies beyond enclosed public places. Thailand provides an interesting case study for estimating such novel legislation since it has recently banned smoking in beaches and in private homes where the smoker could endanger the health of other residents such as children [56,57].

In conclusion, our findings support the need for implementing comprehensive smoke-free policies beyond high-income settings. Despite the less favorable conditions in MICs, smoke-free legislation can potentially bring about similarly positive changes in infant health as in wealthier countries. Extending smoke-free policies to other countries is essential as still only 22% of the world's population is protected by a comprehensive smoke-free law [5]. In MICs, strong tobacco control policy could counterbalance the increasing influence of the tobacco industry [5]. Further, expanding smoke-free policies could help to reach SDG 3.2. which otherwise might not be achieved in MICs [58]. Clean air should not be the privilege for children in HICs, but the right of all children around the world [6].

## Appendix Supplementary materials

Image, application 1

Image, application 2
