# Supplementary material for: Investigating the effects of comprehensive smoke-free legislation on neonatal and infant mortality in Thailand using the synthetic control method
Source: eClinicalMedicine. 2020 Oct 2;27:100560. doi: 10.1016/j.eclinm.2020.100560 (PMC7533363; doi:10.1016/j.eclinm.2020.100560)
Supplement: Supplementary file 2 [file mmc2.docx]

**Supplementary Appendix**

Figure S1 – Policy context of comprehensive smoke-free legislation and observational period of this study


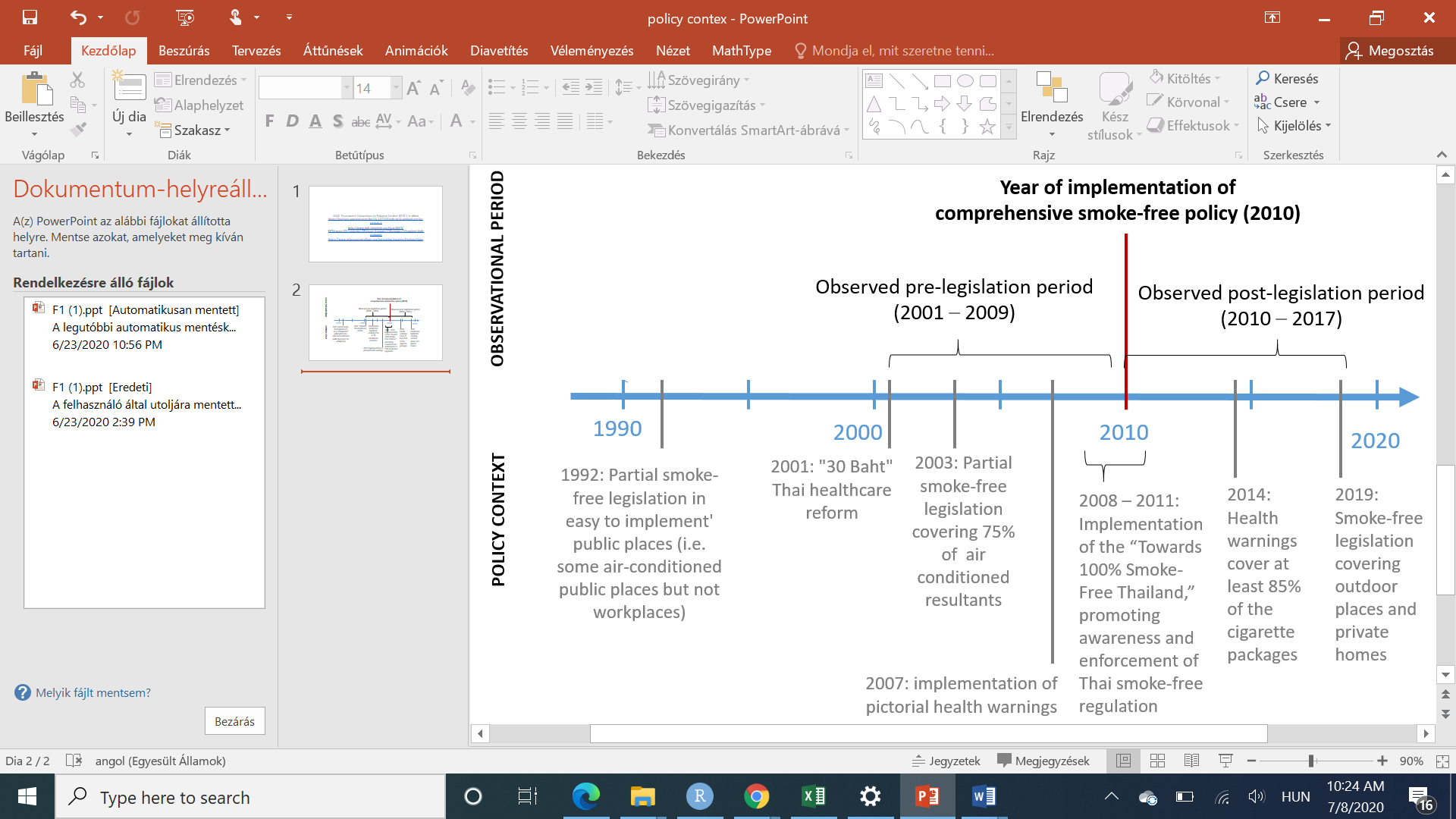


**Table S1 – Control countries included in the donor pools**

|  | **Main analysis** | **Sensitivity analysis 1: Upper-middle income countries** | **Sensitivity analysis 2: East Asian and Pacific** | **Sensitivity analysis 3: without Malaysia** | **Sensitivity analysis 4: countries without any smoke-free legislation** |
| --- | --- | --- | --- | --- | --- |
| Number of countries | 49 | 28 | 12 | 48 | 18 |
| List of Countries | Algeria, Armenia, Azerbaijan, Bangladesh, Belarus, Belize, Bhutan, Botswana, Cabo Verde, Cameroon, China, Cote d'Ivoire, Dominica, Dominican Republic, Equatorial Guinea, Fiji, Gabon, Georgia, Ghana, Grenada, Guyana, India, Indonesia, Iraq, Jordan, Kazakhstan, Kenya, Kiribati, Kyrgyz Republic, Lesotho, Malaysia, Maldives, Mauritania, Mauritius, Mexico, Moldova, Morocco, Nicaragua, Niger, Paraguay, Philippines, Samoa, Sao Tome and Principe, Serbia, South Africa, Sri Lanka, St. Lucia, St. Vincent and the Grenadines, Tonga | Algeria, Armenia, Azerbaijan, Belarus, Belize, Botswana, China, Dominica, Dominican Republic, Fiji, Gabon, Equatorial Guinea, Grenada, Guyana, Iraq, Jordan, Kazakhstan, St. Lucia, Maldives, Mexico, Mauritius, Malaysia, Paraguay, Serbia, Tonga, St. Vincent and the Grenadines, Samoa, South Africa | China, Fiji, Indonesia, Japan, Kiribati, Malaysia, Philippines, Samoa, Timor-Leste, Tonga, Vanuatu, Vietnam | Algeria, Armenia, Azerbaijan, Bangladesh, Belarus, Belize, Bhutan, Botswana, Cabo Verde, Cameroon, China, Cote d'Ivoire, Dominica, Dominican Republic, Equatorial Guinea, Fiji, Gabon, Georgia, Ghana, Grenada, Guyana, India, Indonesia, Iraq, Jordan, Kazakhstan, Kenya, Kiribati, Kyrgyz Republic, Lesotho, Maldives, Mauritania, Mauritius, Mexico, Moldova, Morocco, Nicaragua, Niger, Paraguay, Philippines, Samoa, Sao Tome and Principe, Serbia, South Africa, Sri Lanka, St. Lucia, St. Vincent and the Grenadines, Tonga | Belarus, Belize, Bosnia and Herzegovina, Botswana, Côte d'Ivoire, Dominica, Equatorial Guinea, Grenada, Kenya, Malaysia, Saint Vincent and the Grenadines, Sao Tome and Principe, South Africa, Sudan, Sweden, Switzerland, Tunisia, Vanuatu |

**Table S2 – Control countries with missing data (for the main analysis)**

| **Country** | **Income level** | **East Asian and Pacific** | **Variables with missing data** |
| --- | --- | --- | --- |
| American Samoa | upper-middle | Yes | Infant mortality, Neonatal mortality, GDP, Health expenditure, Clean cooking, Hospital beds, CO_2_, Female primary education completion rate, Fertility rate, Openness to trade |
| Bolivia | lower-middle | No | Female primary education completion rate |
| Bosnia and Herzegovina | upper-middle | No | GDP, Health expenditure |
| Cuba | upper-middle | No | GDP, Health expenditure |
| Djibouti | lower-middle | No | GDP |
| Egypt, Arab Rep. | lower-middle | No | CO_2_ |
| Korea, Dem. People’s Rep. | low | Yes | GDP, Health expenditure, CO_2_ , Openness to trade |
| Korea, Rep. | high | Yes | CO_2_ |
| Macao SAR, China | high | Yes | Infant mortality, Neonatal mortality, Health expenditure, CO_2_, Clean cooking, Hospital beds |
| Micronesia, Fed. Sts. | lower-middle | Yes | CO_2_ , Female primary education completion rate |
| Montenegro | upper-middle | No | Female primary education completion rate |
| Myanmar | lower-middle | Yes | Openness to trade |
| New Caledonia | high | Yes | Neonatal mortality, Infant mortality, GDP, Health expenditure, Clean cooking, Hospital beds, Female primary education completion rate, Openness to trade |
| Northern Mariana Islands | high | Yes | Neonatal mortality, Infant mortality, GDP, Health expenditure, Clean cooking, Hospital beds, CO2 , Female primary education completion rate, Fertility rate, Openness to trade |
| Palau | high | Yes | Female primary education completion rate |
| Singapore | high | Yes | Female primary education completion rate |
| Solomon Islands | lower-middle | Yes | Female primary education completion rate |
| Sudan | lower-middle | No | Female primary education completion rate |
| Tuvalu | upper-middle | Yes | Fertility rate, Openness to trade |

Abbreviations: GDP= Gross domestic product; CO_2=_ Carbon dioxide

**Table S3 – Variable weights for creating the synthetic control countries in the main analysis**

| **Variables** | **Variable weights**  **(for neonatal mortality)** | **Variable weights**  **(for infant mortality)** |
| --- | --- | --- |
| Infant mortality | 0·15 | 0·88 |
| Neonatal mortality | 0·43 | <0·01 |
| GDP (PPP) | 0·01 | 0·03 |
| Health expenditure (PPP) | 0·06 | 0·01 |
| Clean cooking (proportion of the population) | 0·09 | 0·01 |
| Hospital beds (per 1,000 people) | 0·10 | 0·01 |
| Drinking water (proportion of the population) | <0·01 | <0·01 |
| CO_2_ (kiloton) | 0·04 | 0·03 |
| Rural population (proportion of the population) | <0·01 | <0·01 |
| Female primary education completion rate (proportion of the population) | 0·06 | 0·01 |
| Fertility rate (average number of children) | 0·01 | 0·01 |
| Openness to trade (exports plus imports / nominal GDP) | 0·05 | 0·01 |

Abbreviations: GDP= Gross domestic product; PPP= Purchasing power parity; CO_2=_ Carbon dioxide

The calculation of root mean squared prediction error (RMPSE) for the pre-legislation period is done by the following equation:

|  | $RMSPE_{Y}=\sqrt{\frac{1}{(2009-2001)}\sum_{t=2001}^{t=2009} \left(Y_{1}^{t}-\sum_{n=2}^{N} W_{n}^{Y}\times Y_{n}^{t}\right)^{2}}$ | (S1) |
| --- | --- | --- |

**Table S4 – Placebo effects for countries in the donor pool in case of neonatal mortality (main analysis)**

|  | **Algeria** | **Armenia** | **Bhutan** | **Dominican Republic** | **Gabon** | **Georgia** | **Ghana** | **Grenada** | **Guyana** | **Indonesia** | **Iraq** | **Jordan** | **Kenya** | **Mauritania** | **Mauritius** | **Morocco** | **Niger** | **Paraguay** | **Philippines** | **Samoa** | **Sao Tome and Principe** | **Serbia** | **South Africa** | **St. Vincent and the Grenadines** | **Thailand** | **Tonga** |
| --- | --- | --- | --- | --- | --- | --- | --- | --- | --- | --- | --- | --- | --- | --- | --- | --- | --- | --- | --- | --- | --- | --- | --- | --- | --- | --- |
| **RMPSE<0.03** | **Yes** | **Yes** | **Yes** | **Yes** | **Yes** | **Yes** | **Yes** | **Yes** | **Yes** | **Yes** | **Yes** | **Yes** | **Yes** | **Yes** | **Yes** | **Yes** | **Yes** | **Yes** | **Yes** | **Yes** | **Yes** | **Yes** | **Yes** | **Yes** | **Yes** | **Yes** |
| **RMPSE<0.019** | **Yes** | **No** | **Yes** | **Yes** | **Yes** | **No** | **Yes** | **No** | **Yes** | **Yes** | **Yes** | **Yes** | **No** | **Yes** | **No** | **Yes** | **Yes** | **Yes** | **Yes** | **Yes** | **Yes** | **No** | **No** | **Yes** | **Yes** | **No** |
| 2001 | -0·20 | -0·29 | -0·07 | 0·07 | -0·01 | 0·04 | 0·11 | 0·10 | -0·01 | -0·03 | -0·05 | -0·03 | -0·26 | 0·02 | 0·26 | -0·07 | 0·18 | -0·17 | -0·05 | 0·01 | 0·01 | -0·36 | -0·28 | -0·17 | -0·25 | -0·67 |
| 2002 | 0·07 | -0·08 | 0·05 | 0·00 | 0·04 | 0·16 | 0·01 | -0·01 | 0·07 | -0·01 | 0·10 | -0·04 | 0·07 | -0·04 | -0·15 | -0·02 | 0·01 | -0·02 | 0·05 | -0·02 | 0·14 | 0·06 | -0·15 | 0·00 | -0·07 | -0·14 |
| 2003 | 0·15 | 0·06 | 0·04 | 0·08 | 0·04 | 0·36 | -0·06 | -0·08 | -0·01 | -0·01 | -0·02 | 0·05 | 0·24 | -0·07 | -0·29 | -0·04 | -0·02 | 0·03 | 0·05 | 0·02 | 0·10 | 0·14 | 0·03 | 0·02 | 0·05 | 0·10 |
| 2004 | 0·06 | 0·20 | 0·03 | 0·13 | -0·04 | 0·37 | -0·04 | -0·12 | 0·05 | 0·13 | -0·03 | 0·07 | 0·14 | 0·09 | -0·17 | 0·04 | -0·08 | 0·10 | -0·02 | -0·05 | -0·05 | -0·06 | 0·19 | -0·02 | 0·11 | 0·06 |
| 2005 | 0·05 | 0·23 | 0·06 | 0·05 | 0·04 | 0·34 | -0·03 | -0·18 | 0·02 | -0·08 | 0·00 | -0·02 | 0·08 | 0·18 | 0·17 | 0·05 | -0·16 | 0·11 | -0·07 | -0·01 | -0·09 | -0·14 | 0·24 | 0·08 | 0·08 | 0·13 |
| 2006 | -0·04 | 0·20 | -0·08 | 0·06 | -0·02 | 0·16 | 0·03 | -0·15 | 0·00 | 0·04 | -0·01 | 0·04 | -0·11 | 0·13 | 0·29 | 0·17 | -0·08 | 0·10 | 0·00 | 0·00 | -0·14 | -0·15 | 0·24 | 0·04 | 0·11 | 0·05 |
| 2007 | -0·01 | 0·08 | -0·04 | 0·10 | -0·01 | -0·06 | 0·05 | -0·02 | -0·02 | 0·07 | -0·01 | -0·04 | -0·23 | 0·09 | 0·23 | 0·18 | -0·05 | 0·07 | 0·04 | 0·02 | -0·15 | -0·07 | -0·03 | 0·07 | -0·05 | 0·00 |
| 2008 | 0·00 | -0·13 | -0·05 | 0·09 | 0·07 | -0·29 | 0·11 | 0·10 | 0·00 | -0·02 | 0·00 | -0·03 | -0·07 | 0·02 | -0·08 | -0·05 | 0·01 | -0·04 | 0·01 | -0·06 | -0·01 | 0·03 | -0·16 | 0·08 | -0·12 | 0·12 |
| 2009 | -0·05 | -0·33 | 0·08 | 0·02 | -0·01 | -0·40 | -0·07 | 0·33 | 0·06 | -0·09 | 0·03 | -0·01 | 0·15 | -0·16 | -0·30 | -0·26 | 0·19 | -0·17 | -0·01 | 0·04 | 0·20 | 0·09 | -0·32 | 0·00 | -0·24 | 0·25 |
| 2010 | -0·11 | -0·50 | 0·13 | -0·05 | -0·04 | -0·62 | -0·29 | 0·68 | 0·07 | -0·21 | 0·05 | -0·08 | 0·34 | -0·25 | -0·31 | -0·55 | 0·31 | -0·23 | 0·08 | -0·15 | 0·42 | 0·16 | -0·54 | -0·10 | -0·28 | 0·46 |
| 2011 | 0·07 | -0·72 | 0·21 | -0·22 | -0·05 | -0·76 | -0·63 | 1·15 | 0·12 | -0·39 | -0·01 | -0·18 | 0·53 | -0·37 | -0·01 | -0·86 | 0·53 | -0·29 | 0·07 | -0·15 | 0·56 | 0·12 | -0·66 | -0·24 | -0·43 | 0·46 |
| 2012 | 0·50 | -0·84 | 0·36 | -0·50 | -0·12 | -0·99 | -1·13 | 1·60 | 0·02 | -0·55 | -0·22 | -0·13 | 0·68 | -0·63 | 0·20 | -1·22 | 0·84 | -0·40 | 0·18 | -0·17 | 0·56 | -0·08 | -0·77 | -0·53 | -0·53 | 0·32 |
| 2013 | 0·94 | -1·10 | 0·41 | -1·02 | -0·07 | -1·26 | -1·61 | 2·14 | -0·05 | -0·73 | -0·31 | -0·24 | 0·91 | -0·78 | 0·36 | -1·60 | 0·91 | -0·52 | 0·31 | -0·17 | 0·47 | -0·15 | -0·69 | -0·78 | -0·66 | 0·23 |
| 2014 | 1·41 | -1·20 | 0·31 | -1·58 | -0·05 | -1·57 | -2·05 | 2·74 | -0·20 | -0·87 | -0·56 | -0·16 | 1·11 | -0·91 | 0·57 | -1·93 | 1·06 | -0·65 | 0·44 | -0·12 | 0·38 | -0·26 | -0·57 | -1·01 | -0·83 | -0·02 |
| 2015 | 1·79 | -1·32 | 0·31 | -2·24 | 0·01 | -1·89 | -2·47 | 3·32 | -0·34 | -0·97 | -0·62 | -0·28 | 1·29 | -1·27 | 0·45 | -2·09 | 1·17 | -0·78 | 0·63 | -0·14 | -0·06 | -0·46 | -0·52 | -1·14 | -1·01 | -0·17 |
| 2016 | 1·96 | -1·51 | 0·42 | -2·70 | 0·04 | -2·13 | -2·93 | 3·74 | -0·43 | -1·13 | -0·90 | -0·20 | 1·42 | -1·27 | 0·21 | -2·47 | 1·25 | -0·90 | 0·70 | -0·27 | -0·42 | -0·61 | -0·36 | -1·22 | -1·19 | -0·22 |
| 2017 | 2·03 | -1·60 | 0·44 | -3·15 | 0·07 | -2·24 | -3·31 | 3·94 | -0·47 | -1·21 | -1·06 | -0·29 | 1·55 | -1·23 | -0·05 | -2·62 | 1·50 | -0·94 | 0·65 | -0·23 | -0·54 | -0·66 | -0·37 | -1·19 | -1·37 | -0·34 |

Abbreviations: RMPSE = root mean squared prediction error

**Table S5 – Placebo effects for countries in the donor pool in case of infant mortality (main analysis)**

|  | **Algeria** | **Armenia** | **Bangladesh** | **Belize** | **Cameroon** | **Cote d'Ivoire** | **Ghana** | **Grenada** | **Guyana** | **India** | **Indonesia** | **Iraq** | **Jordan** | **Mauritius** | **Mexico** | **Nicaragua** | **Philippines** | **Samoa** | **Serbia** | **Thailand** |
| --- | --- | --- | --- | --- | --- | --- | --- | --- | --- | --- | --- | --- | --- | --- | --- | --- | --- | --- | --- | --- |
| **RMPSE<0.03** | **Yes** | **Yes** | **Yes** | **Yes** | **Yes** | **Yes** | **Yes** | **Yes** | **Yes** | **Yes** | **Yes** | **Yes** | **Yes** | **Yes** | **Yes** | **Yes** | **Yes** | **Yes** | **Yes** | **Yes** |
| **RMPSE<0.019** | **Yes** | **Yes** | **Yes** | **Yes** | **Yes** | **No** | **Yes** | **Yes** | **Yes** | **Yes** | **Yes** | **Yes** | **Yes** | **No** | **No** | **Yes** | **Yes** | **No** | **No** | **Yes** |
| 2001 | -0·35 | 0·03 | 0·07 | -0·06 | 0·12 | 0·01 | -0·03 | 0·03 | 0·07 | 0·01 | 0·08 | 0·04 | -0·15 | 0·44 | 0·25 | -0·09 | -0·17 | 0·24 | 0·01 | -0·12 |
| 2002 | 0·10 | 0·00 | -0·07 | -0·10 | 0·01 | -0·01 | -0·22 | 0·00 | -0·04 | -0·02 | 0·00 | 0·08 | -0·11 | -0·13 | 0·13 | 0·02 | 0·08 | 0·15 | 0·25 | 0·12 |
| 2003 | 0·21 | 0·08 | -0·08 | -0·03 | -0·10 | -0·04 | -0·21 | -0·07 | -0·11 | -0·07 | -0·07 | -0·04 | 0·04 | -0·36 | -0·04 | 0·11 | 0·19 | 0·06 | 0·09 | 0·21 |
| 2004 | 0·18 | 0·10 | 0·02 | 0·09 | -0·06 | -0·19 | -0·20 | -0·04 | -0·17 | -0·18 | 0·05 | -0·08 | -0·02 | -0·33 | -0·22 | -0·20 | -0·06 | -0·02 | -0·19 | -0·03 |
| 2005 | 0·08 | 0·03 | 0·01 | 0·11 | 0·17 | -0·21 | -0·03 | -0·05 | -0·14 | 0·00 | -0·07 | -0·05 | 0·03 | -0·02 | -0·27 | 0·17 | 0·06 | -0·16 | -0·35 | 0·11 |
| 2006 | -0·10 | -0·02 | 0·09 | 0·07 | 0·05 | 0·16 | 0·10 | -0·15 | -0·04 | 0·14 | -0·05 | -0·08 | -0·02 | 0·23 | -0·27 | 0·09 | 0·05 | -0·29 | -0·31 | 0·01 |
| 2007 | -0·07 | 0·03 | 0·02 | 0·05 | 0·22 | 0·44 | 0·07 | -0·10 | 0·25 | 0·17 | -0·02 | -0·02 | 0·04 | 0·36 | -0·08 | 0·05 | -0·06 | -0·31 | -0·20 | -0·15 |
| 2008 | -0·07 | 0·00 | 0·00 | 0·00 | 0·12 | -0·04 | -0·01 | 0·08 | -0·07 | 0·00 | 0·06 | 0·05 | 0·09 | 0·07 | 0·07 | -0·05 | -0·04 | -0·34 | 0·09 | -0·24 |
| 2009 | 0·06 | 0·10 | -0·08 | -0·12 | -0·06 | 0·49 | -0·38 | 0·29 | -0·06 | 0·03 | 0·03 | 0·11 | 0·10 | -0·29 | 0·37 | -0·13 | -0·12 | 0·55 | 0·27 | -0·39 |
| 2010 | 0·39 | -0·11 | -0·25 | -0·34 | -0·99 | 1·37 | -0·96 | 0·58 | 0·98 | 0·33 | 0·01 | 0·15 | 0·10 | -0·73 | 0·49 | -0·15 | 0·17 | -0·54 | 0·36 | -0·65 |
| 2011 | 0·83 | -0·50 | -0·40 | -0·65 | -1·63 | 1·78 | -1·53 | 1·04 | 0·96 | 0·02 | 0·01 | 0·17 | 0·11 | -0·87 | 0·54 | -0·10 | 0·38 | -0·55 | 0·41 | -0·76 |
| 2012 | 1·37 | -0·98 | -0·69 | -0·94 | -2·13 | 1·58 | -2·20 | 1·52 | 0·70 | -0·32 | 0·04 | 0·27 | 0·07 | -1·04 | 0·60 | -0·06 | 0·57 | -0·60 | 0·29 | -0·91 |
| 2013 | 2·05 | -1·31 | -1·00 | -1·32 | -2·61 | 1·55 | -2·79 | 2·12 | 0·55 | -0·61 | 0·18 | 0·11 | 0·09 | -1·24 | 0·63 | -0·01 | 0·81 | -0·57 | 0·14 | -1·11 |
| 2014 | 2·61 | -1·72 | -1·30 | -1·67 | -2·79 | 1·83 | -3·51 | 2·78 | 0·50 | -1·00 | 0·39 | 0·04 | 0·05 | -1·52 | 0·53 | 0·06 | 0·99 | -0·58 | -0·01 | -1·34 |
| 2015 | 3·07 | -2·17 | -1·76 | -2·13 | -3·31 | 1·84 | -3·95 | 3·38 | 0·10 | -1·35 | 0·57 | -0·10 | -0·03 | -1·87 | 0·28 | 0·18 | 1·18 | -0·50 | -0·31 | -1·56 |
| 2016 | 3·45 | -2·42 | -2·00 | -2·48 | -3·21 | 1·95 | -4·37 | 3·85 | 0·09 | -1·60 | 0·80 | -0·21 | 0·03 | -2·57 | 0·09 | 0·19 | 1·33 | -0·55 | -0·52 | -1·83 |
| 2017 | 3·77 | -2·75 | -2·31 | -2·75 | -3·04 | 2·07 | -4·61 | 4·31 | 0·10 | -1·81 | 0·89 | -0·36 | -0·06 | -3·15 | -0·35 | 0·19 | 1·25 | -0·40 | -0·68 | -1·96 |

Abbreviations: RMPSE = root mean squared prediction error

**Table S6 – Country weights in the synthetic control countries when donor pool includes upper-middle-income countries only**

| Country* | Country weights | |
| --- | --- | --- |
|  | Neonatal mortality | Infant mortality |
| Malaysia | 0·414 | 0·444 |
| China | 0·204 | 0·314 |
| Serbia | 0·193 | – |
| Mauritius | 0·131 | 0·129 |
| Equatorial Guinea | 0·058 | – |
| Armenia | – | 0·047 |
| Guyana | – | 0·041 |
| Iraq | – | 0·025 |

*This table contains only those control countries that contributed to the constitution of the synthetic control country with a larger than 0 weight.

**Table S7 – Country weights in the synthetic control countries when donor pool contains only countries from the East Asian and Pacific region**

| **Country*** | **Country weights** | |
| --- | --- | --- |
|  | **Neonatal mortality** | **Infant mortality** |
| Malaysia | 0·431 | 0·475 |
| China | 0·320 | 0·279 |
| Vanuatu | 0·249 | – |
| Vietnam | – | 0·202 |
| Indonesia | – | 0·039 |
| Philippines | – | 0·004 |

*This table contains only those control countries that contributed to the constitution of the synthetic control country with a larger than 0 weight.

**Table S8 – Country weights in the synthetic control countries when donor pool includes middle-income countries and excludes Malaysia that received the highest weight in the main analysis**

| **Country*** | **Country weights** | |
| --- | --- | --- |
|  | **Neonatal mortality** | **Infant mortality** |
| Nigeria | 0·253 | – |
| Belarus | 0·223 | 0·074 |
| Mauritius | 0·218 | 0·524 |
| Serbia | 0·201 | – |
| Morocco | 0·062 | – |
| China | 0·034 | 0·007 |
| Bangladesh | 0·007 | – |
| Maldives | – | 0·221 |
| Sri Lanka | – | 0·174 |
| China | – | 0·007 |
| Fiji | – | 0·001 |

*This table contains only those control countries that contributed to the constitution of the synthetic control country.

**Figure S2 – Trends in neonatal and infant mortality: Thailand versus the synthetic control country (2001–2017) after excluding Malaysia that contributed the heaviest weight to the synthetic control country in the main analysis**


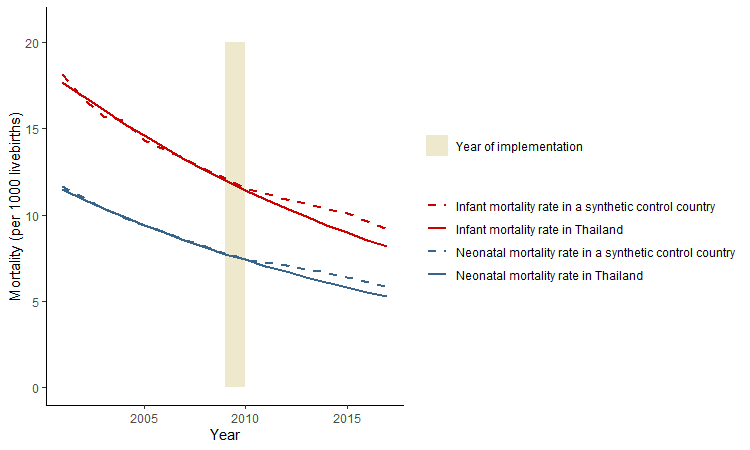


**Table S9 – Country weights in the synthetic control countries when donor pool consists middle-income countries that did not introduce any smoke-free legislation**

| **Country*** | **Country weights** | |
| --- | --- | --- |
|  | **Neonatal mortality** | **Infant mortality** |
| Belarus | 0·753 | 0·875 |
| Côte d'Ivoire | 0·153 | 0·088 |
| Malaysia | 0·094 | 0·001 |
| Dominica | – | 0·014 |
| South Africa | – | 0·012 |
| Vanuatu | – | 0·006 |
| Kenya | – | 0·001 |
| Sao Tome and Principe | – | 0·001 |

*This table contains only those control countries that contributed to the constitution of the synthetic control country with a larger than 0 weight.

**Figure S3 – Trends in neonatal and infant mortality: Thailand versus the synthetic control country (2001–2017) when donor pool consists middle-income countries that did not introduce any smoke-free legislation**

**
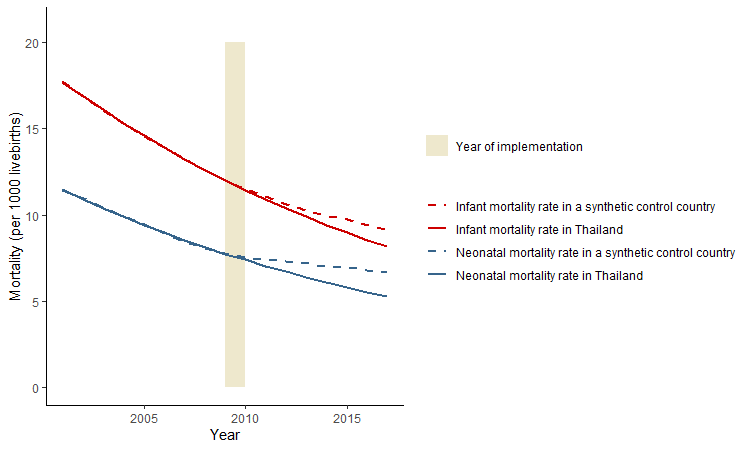
**

**Table S10 – Country weights in the synthetic control countries after missing value imputation**

| **Country*** | **Country weights** | |
| --- | --- | --- |
|  | **Neonatal mortality** | **Infant mortality** |
| Mauritius | 0·369 | 0·487 |
| Malaysia | 0·181 | 0·487 |
| Maldives | 0·178 | – |
| Sri Lanka | 0·168 | 0·076 |
| St. Lucia | 0·062 | – |
| China | 0·028 | 0·028 |
| Bangladesh | 0·001 | – |
| Nicaragua | – | 0·261 |
| Moldova | 0·249 | 0·123 |
| Bhutan | – | 0·025 |

*This table contains only those control countries that contributed to the constitution of the synthetic control country with a larger than 0 weight.

**Figure S4 – Trends in neonatal and infant mortality: Thailand versus the synthetic control country (2001–2017) after missing value imputation**

**
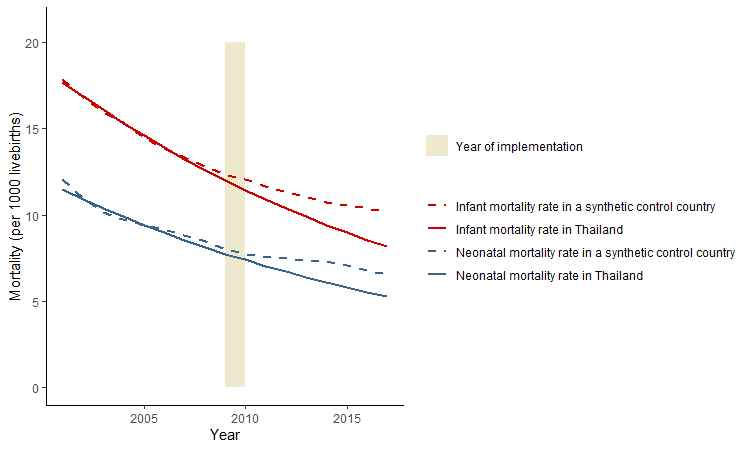
**

**Table S11 – Country weights in the synthetic control countries when cigarette consumption was included as a predictor variable**

| **Country*** | **Country weights** | |
| --- | --- | --- |
|  | **Neonatal mortality** | **Infant mortality** |
| Malaysia | 0·578 | 0·533 |
| Armenia | 0·230 | 0·253 |
| China | 0·122 | 0·126 |
| Bangladesh | 0·066 | 0·050 |
| Indonesia | 0·003 | – |
| Moldova | 0·001 | 0·017 |

*This table contains only those control countries that contributed to the constitution of the synthetic control country with a larger than 0 weight.

**Figure S5 – Trends in neonatal and infant mortality: Thailand versus the synthetic control country (2001–2017) when cigarette consumption was included as a predictor variable**

**
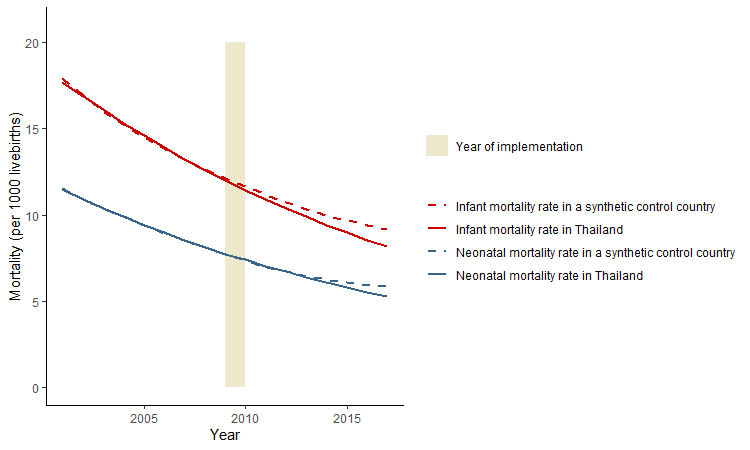
**
